# Celastrol Activates HSF1 to Enhance Regulatory T Cells Function and Ameliorate Intestinal Inflammation

**DOI:** 10.3390/biom16010062

**Published:** 2025-12-31

**Authors:** Kibrom M. Alula, Colm B. Collins, Tom T. Nguyen, Carol M. Aherne, Paul Jedlicka, Edwin F. de Zoeten

**Affiliations:** 1 Mucosal Inflammation Program, University of Colorado School of Medicine, Aurora, CO 80045, USA; kibrom.alula@cuanschutz.edu (K.M.A.); colm.collins@ucd.ie (C.B.C.); tom.nguyen@cuanschutz.edu (T.T.N.); carol.aherne@ucd.ie (C.M.A.); 2 Department of Pediatrics, Division of Gastroenterology, Hepatology and Nutrition, University of Colorado School of Medicine, Aurora, CO 80045, USA; 3 Department of Anesthesiology, University of Colorado School of Medicine, Aurora, CO 80045, USA; 4 Department of Pathology, University of Colorado School of Medicine, Aurora, CO 80045, USA; paul.jedlicka@cuanschutz.edu

**Keywords:** inflammatory bowel disease, heat shock factor 1, celastrol, regulatory T cell

## Abstract

Inflammatory Bowel Disease (IBD) is a chronic inflammatory condition resulting from dysregulation of the intestinal immune system. CD4^+^FoxP3^+^ regulatory T cells (Tregs) play a crucial role in regulating this immune response. The heat shock response (HSR) regulates the inflammatory cascade, preventing misfolding of proteins and regulating immune responses. We have previously shown that Heat Shock Factor 1 (HSF1), the master regulator of the HSR, regulates Tregs in inflammation. Based on this finding, we hypothesized that targeting HSF1 with celastrol, a pentacyclic triterpenoid that activates HSF1, would activate Treg cells and ameliorate intestinal inflammation. To test this, we investigated the impact of celastrol on Tregs both in vitro and in vivo, evaluating its efficacy in HSF1^fl/fl^-CD4^cre^ mice, and in two murine models of IBD: the adoptive transfer colitis, and TNF^ΔARE+/−^ ileitis. Our results demonstrate that celastrol activates HSF1 in Tregs, enhances Treg suppressive function, increases Treg populations in vivo, and ameliorates intestinal inflammation.

## 1. Introduction

Inflammatory bowel diseases (IBDs), including Crohn’s disease and ulcerative colitis, are chronic immune-mediated disorders affecting approximately 3.1 million individuals in the United States, with nearly one-third of cases diagnosed in children [1]. Despite advances in therapies, such as anti-tumor necrosis factor-alpha (TNFα) agents, fewer than 50% of patients achieve sustained remission [2,3]. Moreover, current FDA-approved therapies for IBD, including TNF inhibitors (e.g., infliximab and adalimumab) [4], IL-23 inhibitors (e.g., mirikizumab, risankizumab), Janus kinase (JAK) inhibitors (e.g., tofacitinib and upadacitinib) [5], focus primarily on broadly suppressing inflammation. However, none of them specifically enhance regulatory CD4^+^FoxP3^+^ T cell (Treg) function, despite continued evidence of their critical role in maintaining mucosal immune tolerance and reducing inflammation. Moreover, standard immunosuppressive treatments like corticosteroids (e.g., prednisone) [6] may further impair Treg stability and function, limiting their long-term efficacy and specificity [7,8,9,10]. As such, the development of therapies that directly target and restore Treg suppressive capacity represents an unmet therapeutic avenue in IBD management.

The pathogenesis of IBD involves complex interactions between host genetics, microbial dysbiosis, and a dysregulated immune response [11]. Tregs are key players in maintaining mucosal immune homeostasis, which suppress pro-inflammatory responses and promote immune tolerance [7]. Impaired Treg function is linked to severe intestinal inflammation, as seen in disorders such as immune dysregulation, polyendocrinopathy, enteropathy, X-linked (IPEX) syndrome [12,13,14]. While some studies have reported a reduction in both Treg number and function in IBD [7,15,16], others have shown that Tregs accumulate in inflamed tissues yet remain functionally defective [17,18,19]. This paradox suggests that enhancing Treg number alone is insufficient, and strategies must also restore their suppressive capacity.

Our recently published work has demonstrated that upregulation of heat shock factor 1 (HSF1) in Tregs supports their function, including IL-10 secretion [20]. HSF1 is the central transcriptional regulator of the Heat Shock Response (HSR), a stress-induced pathway that promotes cellular protection and anti-inflammatory signaling [21]. Moreover, we demonstrated that TGF-β enhances HSF1 binding to its DNA recognition motif, the heat shock response element (HSE) [22], and we have shown, by chromatin immunoprecipitation, that HSF1 binds to the FoxP3 promoter [20] and that HSF1 is activated by T cell receptor (TCR) stimulation and diverse stressors [23,24,25,26,27].

Building on these findings, we explored HSF1 as a therapeutic target to enhance Treg function in IBD. Pharmacologic HSF1 activators, including celastrol and HSF1A, are known to increase nuclear HSF1 levels and have been shown to improve Treg-associated immune regulation [28,29]. Celastrol, a pentacyclic triterpenoid derived from *Tripterygium wilfordii* (Thunder God Vine), activates HSF1 in a manner like heat shock, inducing phosphorylation and DNA binding [28]. Consequently, it has been shown to reduce disease severity in DSS-induced colitis in mice [30] and to inhibit inflammatory mediators such as NF-κB, endoplasmic reticulum Ca^2+^ ATPase, and myeloid differentiation factor 2 (MD2) in models of rheumatoid arthritis [31]. Therefore, by targeting CD4^+^FoxP3^+^ Tregs, celastrol may favor regulatory responses that attenuate intestinal inflammation [32]. Although higher doses (>3 mg/kg) of celastrol have been associated with adverse effects [33,34], our study provides proof-of-concept that targeting HSF1 can enhance intestinal immune responses when using doses well below those reported to be toxic (<2 mg/kg in vivo and 100 nM in vitro) in rodent studies.

## 2. Materials and Methods

### 2.1. Ethics Declaration

All animal procedures were conducted in compliance with the guidelines and regulations of the Institutional Animal Care and Use Committees (IACUC: 00209) at the University of Colorado Anschutz Medical Campus (AAALAC: 00235).

### 2.2. Animal Models

Male C57BL/6J (000664), HSF1^−^/^−^ (C;129-*Hsf1^tm1Ijb^*/J; 012052), Rag1^−/−^ (002216), and CD4^Cre^ (017336) mice were obtained from Jackson Laboratories (Bar Harbor, ME, USA). The TNF^ΔARE/+^ strain (B6.129S-Tnf^tm2GKl^/Jarn; MGI:3720980) was established through successive backcrossing of heterozygous TNF^ΔARE/+^ mice (on a mixed background [35]) to C57BL/6J mice [36] and maintained under specific-pathogen-free (SPF) conditions. Experimental animals were either heterozygous for the ΔARE mutation (TNF^∆ARE/+^) or homozygous wild-type (WT) littermates, which served as controls. Maximum transmural ileitis occurs in TNF^∆ARE/+^ mice at 8–12 weeks of age, and thus all experiments were conducted using animals within this age range. To generate T cell-specific deletion of HSF1, HSF1^loxP/loxP64^ mice were crossed with CD4^Cre^ mice, resulting in conditional HSF1 knockout in CD4^+^ T cells. All mice were maintained under specific-pathogen-free conditions and routinely screened negative for *Helicobacter* species, protozoa, and helminths [20].

### 2.3. Isolation of Cells from Spleen and Lamina Propria

Spleens were harvested from C57BL/6J mice and placed in ice-cold RPMI 1640 medium, supplemented with 2% fetal bovine serum (FBS) and 1% penicillin-streptomycin. Single-cell suspensions were prepared by mechanically disrupting the spleens by gently pressing the tissue against 70 μm cell strainer with a sterile plunger. Then, Red blood cells (RBCs) were lysed using 150 mM ACK lysis buffer for 3 min at room temperature, followed by quenching with excess RPMI 1640 medium. Cells were then centrifuged (5 min at 1700 rpm) and resuspended in complete RPMI 1640 culture medium. Similarly, T cells were isolated from colonic and ileal lamina propria as previously described [20].

### 2.4. Treg Conversion Assay

Naïve CD4^+^CD25^Neg^ T cells were isolated from the spleens of WT mice using the EasySep^TM^ Mouse CD25 Regulatory T Cell Positive Selection Kit II (18782, Stem Cell Technologies, Vancouver, BC, Canada). Purified cells were cultured under Treg-polarizing conditions in complete RPMI-1640 medium supplemented with 10% FBS, 100 IU/mL penicillin, and 100 µg/mL streptomycin (15140-122, gibco, Waltham, MA, USA). Cells were stimulated with plate-bound anti-mouse CD3 (1 µg/mL, 100302, BioLegend, San Diego, CA, USA), soluble anti-mouse CD28 (1 µg/mL, 102102, BioLegend, San Diego, CA, USA), murine IL-2 (5 IU/mL, 212-12, PEPROTECH, Cranbury, NJ, USA), and TGF-β1 (5 ng/mL, 100-21C, PEPROTECH, Cranbury, NJ, USA). To assess the effect of celastrol (C0869, Millipore Sigma, Burlington, MA, USA), cells were co-incubated with DMSO (vehicle) or celastrol (100 nM) for 72 h in RPMI-1640 medium during the conversion. At the end of the incubation, flow cytometry was performed to analyze FoxP3 and IL-10 expressions, which indicate the conversion of naïve CD4^+^CD25^−^ T cells into Tregs.

### 2.5. Luciferase Assay

Early-passage Jurkat cells were seeded at a density of 5 × 10^5^ cells per well in 6-well plates and transfected using Lipofectamine LTX with either an HSE-luciferase reporter constructs (Promega, Madison, WI, USA, E3751) or a FoxP3 CNS2-luciferase reporter construct (provided by M. Tone, University of Pennsylvania), along with Renilla luciferase as an internal control (2.5 µg total DNA per well). After 24 h, cells were replated into 96-well plates at 2.5 × 10^5^ cells per well and treated for an additional 24 h under the following conditions: recombinant human TGF-β (10ng/mL; 7754-BH, R&D Systems, Minneapolis, MN, USA), anti-human CD3/CD28 Dynabeads (8ml/well; 11131D, Invitrogen, Waltham, MA, USA), their combination, or celastrol (100 nM). Additional experimental conditions included heat shock stress (56 °C for 6 or 24 h) and treatment with PMA (20 ng/mL) plus Ionomycin (1 µg/mL) to simulate T cell activation. Following treatment, cells were harvested, centrifuged, and lysed in 25 µL passive lysis buffer (Promega). Luciferase activities were measured using the Dual-Luciferase^®^ Reporter Assay System and read on a GloMax^®^-Multi Microplate Multimode Reader (Promega). Luciferase signal was normalized to Renilla to account for transfection efficiency. HSF1 activity was assessed by luciferase expression from the HSE reporter under baseline, heat shock, and celastrol-treated conditions. Additionally, transcriptional activation of the FoxP3 CNS2 enhancer by HSF1 was quantified under unstimulated, PMA/Ionomycin, and celastrol-treated conditions.

### 2.6. Western Blotting

Nuclear and cytoplasmic protein extracts of splenocytes were obtained 6 h post-conversion using the NE-PER Nuclear and Cytoplasmic Extraction Kit (78833, Thermo Scientific, Rockford, IL, USA) following the manufacturer’s protocol. Protein concentrations were determined using the bicinchoninic acid (BCA) assay (Pierce BCA Protein Assay Kit, 23225, Thermo Scientific, Rockford, IL, USA) according to the manufacturer’s instructions. For Western blot analysis, equal amounts of protein were resolved under both denaturing and non-denaturing conditions on 4–15% gradient precast gels (4561086, Bio-Rad, Hercules, CA, USA) and transferred onto PVDF membranes (1620174, Bio-Rad, Hercules, CA, USA). Membranes were blocked in 5% non-fat dry milk in TBST for 1 h at room temperature and incubated overnight at 4 °C with primary antibodies: HSF1 (4356S, 1:500, Cell Signaling, Danvers, MA, USA), TBP (ab818, 1:1000, Abcam, Cambridge, UK), and β-actin (664804, 1:10,000, BioLegend, San Diego, CA, USA). The next day, membranes were washed and incubated with corresponding HRP-conjugated secondary antibodies: goat anti-rat (sc-2006, 1:1000, Santa Cruz, Dallas, TX, USA) for HSF1 and β-actin, and goat anti-mouse (#31430, 1:1000, Thermo Scientific, Waltham, MA, USA) for TBP, each for 1 h at room temperature. TBP served as a loading control for nuclear extracts, while β-actin was used as a cytoplasmic loading control. Blots were developed using the Clarity Western ECL Substrate (170-5060, Bio-Rad, Hercules, CA, USA) for 5 min and imaged using the Bio-Rad ChemiDoc™ MP system. Band intensities (integrated density) were quantified using Adobe Photoshop 2025, and values were normalized to the corresponding housekeeping protein.

### 2.7. Suppression Assay

Treg suppression assays were conducted following previously described methods [20,37]. In brief, CD4^+^CD25^+^ Tregs and CD4^+^CD25^Neg^ T cells were isolated from murine (WT and HSF1^fl/fl^-CD4^cre^) splenocytes using the EasySep^TM^ Mouse CD4 T cell isolation kit (19852A, STEMCELL Technologies, Vancouver, BC, Canada) and CD25 Regulatory T Cell Positive Selection Kit II (18782, Stem Cell Technologies, Vancouver, BC, Canada). CellTrace Violet-labeled (C34557, Invitrogen, Waltham, MA, USA) CD4^+^CD25^Neg^ WT and HSF1^fl/fl^-CD4^cre^ effector T cells at a concentration of 5 × 10^5^ cells per well were stimulated with anti-CD3 monoclonal antibody (1ug/mL) in the presence of 7.5 × 10^5^ irradiated syngeneic antigen-presenting cells (APC). Prior to irradiation, the APCs were isolated from splenocytes of WT mice using the mouse CD90.2 positive selection kit (18951, Stem Cell Technologies, Vancouver, BC, Canada). CD4^+^CD25^Neg^ T cells were converted to Tregs with or without 100 nM celastrol. Then, these converted Tregs were used at decreasing dilutions for suppressing Teff cells. Suppression of proliferation was determined by assessing the division profile of effector cells after 72 h using flow cytometry.

### 2.8. Murine Colitis Model Using CD45RB^hi^ Adoptive Transfer

The adoptive transfer colitis model involves transferring naïve CD4^+^CD45RB^hi^ T cells into lymphogenic recipients, leading to uncontrolled T cell expansion and effector-driven colonic inflammation due to insufficient Treg-mediated regulation [38]. To investigate the effect of celastrol in a Treg-dependent model of colitis, we used the CD4^+^CD45RB^hi^ adoptive transfer model in immunodeficient B6.RAG1^−^/^−^ mice. CD4^+^ T cells were isolated from 8 to 12-week-old C57BL/6J donor mice using a CD4-negative selection kit (19852A, Stem Cell Technologies, Vancouver, BC, Canada), and the CD4^+^CD25^−^ fraction was further sorted by FACS (BD FACSAria™; BD Biosciences, Milpitas, CA, USA) to obtain CD4^+^CD45RB^hi^ cells. A total of 1 × 10^6^ CD4^+^CD45RB^hi^ cells were intraperitoneally injected into 8–12-week-old B6.RAG1^−^/^−^ recipient mice. After two weeks, colitis symptoms developed (e.g., weight loss), ALZET osmotic pumps (14-day, Model 1002, DURET Corporation, Cupertino, CA, USA) were implanted subcutaneously to deliver either celastrol (2 mg/kg in DMSO) or vehicle (1× PBS/DMSO) at a rate of 0.24 µL/hour. Then, colon tissues were collected for flow cytometry and histology.

### 2.9. TNF^ΔARE^ Model of Ileitis

The TNF^ΔARE^ model develops spontaneous chronic ileitis due to a deletion in the AU-rich element of the TNF gene, resulting in constitutive TNF overproduction and innate-driven inflammation resembling Crohn’s-like ileitis [39]. To assess the effects of celastrol on ileal inflammation, TNF^ΔARE/+^ mice (8–12-week-old) were implanted subcutaneously with ALZET osmotic pumps (Model 1002, DURET Corporation, Cupertino, CA, USA) containing either celastrol (2 mg/kg in DMSO) or vehicle (1× PBS or DMSO) for two weeks. Mice were sacrificed at 10–14 weeks of age, and ileal tissues were collected for histological analysis, including H&E staining and histological scoring, and flow cytometry. For the flow cytometry, CD4^+^ and CD8^+^ T-cell subsets were defined by CD44 and CD62L expression. Naïve T cells were identified as CD44^+^CD62L^Neg^, effector T cells as CD44^+^CD62L^Neg^, and central memory T cells as CD44^+^CD62L^+^. These gating definitions were applied uniformly in all analyses.

### 2.10. Immunohistochemistry

Immunohistochemistry (IHC) was performed on adoptive transfer-induced colitis mice, and ileal tissues from TNF^ΔARE^ mice treated with either vehicle (1× PBS, DMSO) or celastrol. At the study endpoint, tissues were excised, flushed with PBS, and fixed in 10% neutral-buffered formalin for 24 h. Fixed tissues were then paraffin-embedded, sectioned (5 μm), mounted on glass slides for staining, and hematoxylin and eosin (H&E)-stained. Stained sections were imaged using a brightfield microscope, and quantification of positively stained cells was performed using ImageJ (version 1.54m) or QuPath software (version v0.6.0). Blinded histological scoring was conducted (P. Jedlicka) to assess immune infiltration, epithelial damage, and tissue remodeling [40]. Differences between treatment groups were analyzed to determine the effect of celastrol on inflammation and immune cell composition in both TNF^ΔARE/+^ and adoptive transfer colitis models.

Briefly, colonic inflammation was evaluated using a graded histologic scoring system encompassing four features: active inflammation (0.5–3), chronic inflammation (0.5–3), villus architecture (1–3), and cross-sectional area involved (0.5–4). Active inflammation was scored based on the severity of neutrophil infiltration, ranging from scattered polymorphonuclear neutrophils (PMNs) (0.5–1) to marked increases with crypt abscesses or epithelial erosion (2–3). Chronic inflammation reflected the degree of mononuclear cell accumulation in the lamina propria, with minimal changes graded at 0.5–1 and widening of intercryptal spaces or separation of crypt bases indicating higher grades (2–3). The percentage of mucosal tissue affected was scored from <1% (0.5) to 76–100% involvement (4). Scores for all categories were summed to generate a total inflammation score, with higher totals indicating more severe disease. For the TNF^ΔARE^ model, histological scoring was performed as described for the colitis model, with the addition of assessing villus architecture. Villus architecture was evaluated based on the degree of villus height reduction, ranging from mild shortening (score 1) to marked atrophy or complete villus loss (score 3).

### 2.11. Flow Cytometry

For flow cytometry, WT splenocytes, colonic and ileal lamina propria (LP) were resuspended in FACS buffer (1× PBS [21-031-CV, Corning, Corning, NY, USA], 2% FBS [A52567-01, Gibco, Waltham, MA, USA], 0.02% sodium azide [71289, Sigma]) and stained with fluorophore-conjugated antibodies against CD4 (Pacific Blue anti-mouse, 100428, BioLegend, San Diego, CA, USA), FoxP3 (PE-Cy7 anti-mouse/rat FoxP3, 25-5773-82, Invitrogen, Waltham, MA, USA), and IL-10 (PE anti-mouse, 505008, BioLegend, San Diego, CA, USA). Live/dead cells were discriminated using a fixable viability dye (Aqua, L34966A, Invitrogen, Waltham, MA, USA). Intracellular FoxP3 staining was performed using the FoxP3/Transcription Factor Staining Kit (eBioscience, Thermo Fisher) according to the manufacturer’s instructions. Data were acquired on a BD FACSCanto II or BD LSRFortessa and analyzed with FlowJo software (version v10; BD Biosciences, Ashland, OR, USA). Gating information has been included in Figure S2.

### 2.12. Cytokine Assessment by Enzyme-Linked Immunosorbent Assay (ELISA)

ELISA was performed on WT splenocyte culture supernatants. Splenocytes were isolated (as described above) and cultured ex vivo in complete RPMI medium at 37 °C with 5% CO_2_. After 24 h, supernatants were collected and stored at −80 °C until analysis. Cytokine quantification was performed using ELISA kits for IL-10 according to the manufacturer’s protocol (Mouse IL-10, 88-7105, Invitrogen, Waltham, MA, USA). Briefly, 96-well plates were coated with capture antibodies, blocked with 5% BSA, and incubated with diluted samples or standards. Detection was achieved using biotinylated secondary antibodies, followed by incubation with streptavidin-HRP and TMB substrate. Absorbance was measured at 450 nm using the SpectraMax iD5 Microplate Reader (Molecular Devices, San Jose, CA, USA), and cytokine concentrations were calculated based on a standard curve. All samples were analyzed in duplicate or triplicate to ensure accuracy and reproducibility.

### 2.13. Statistics

Statistical analyses were conducted using either the Student’s *t*-test, one-way ANOVA, or two-way ANOVA with Tukey’s multiple comparison test when applicable. Graphs were generated using GraphPad Prism software (version 10.4.2), and the data are presented as means ± SEM. Statistical significance was determined at a threshold of *p* < 0.05.

## 3. Results

### 3.1. Celastrol Enhances Treg Differentiation and IL-10 Production in an HSF1-Dependent Manner

**Figure 1:**
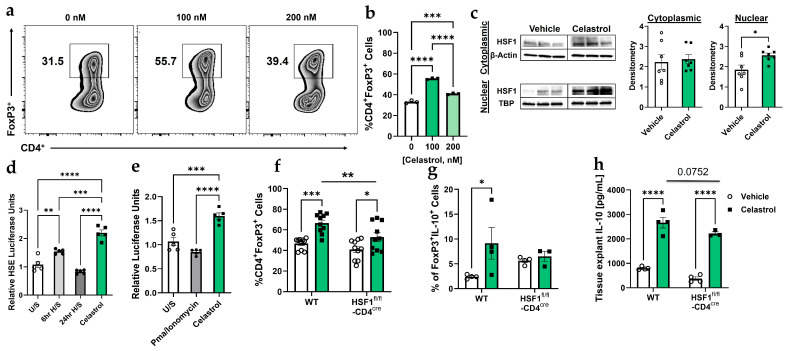
Celastrol enhances Treg functionality in an HSF1-dependent manner. (**a**) Flow cytometry analysis showing the abundance of Foxp3^+^ Treg cells in wild-type (WT) splenocytes treated with increasing concentrations of Celastrol (n = 3). (**b**) Quantification of Treg populations from (**a**), displayed as a bar graph (n = 3). (**c**) Representative of Western blot analysis of HSF1 protein expression in Tregs post-conversion (cytoplasmic and nuclear fractions), comparing Vehicle and 100 nM Celastrol treatment. Densitometric quantification of HSF1 expression was performed using Adobe Photoshop 2025 (n = 7) by normalizing (dividing) each value by its corresponding value of the housekeeping gene. (**d**) Luciferase assay in Jurkat cells assessing HSF1 binding to the heat shock element (HSE) DNA binding site under unstimulated (U/S) conditions, heat shock treatment (56 °C for 6 and 24 h), and 100 nM Celastrol treatment (n = 5). (**e**) FoxP3 promoter activation (CNS2-Luc) in Jurkat cells in response to HSF1 binding under unstimulated (U/S) conditions, 20 ng/mL PMA and 1 ug/mL Ionomycin, and 100 nM Celastrol treatments (n = 5). (**f**) Flow cytometry analysis of CD4^+^Foxp3^+^ Treg populations in WT and HSF1^fl/fl^-CD4^cre^ splenocytes treated with Vehicle (1× PBS) or 100 nM Celastrol (n = 10). (**g**) Flow cytometry analysis of CD4^+^Foxp3^+^IL-10^+^ Treg populations in WT and HSF1^fl/fl^-CD4^cre^ splenocytes treated with Vehicle (1× PBS) or 100 nM Celastrol (n = 4). (**h**) ELISA quantification of IL-10 levels in spleen tissue explants treated with Vehicle or 100 nM Celastrol (n = 4). Results are presented as individual mice ± SEM. Statistical significance was determined using one-way ANOVA (**b**,**d**,**e**), and two-way ANOVA (**f**,**g**,**h**) followed by Tukey’s multiple comparison test (* *p* < 0.05, ** *p* < 0.01, *** *p* < 0.001, **** *p* < 0.0001). Absolute number of cells shown in Figure S3A.

To investigate the effect of celastrol in Treg induction and function, we analyzed CD4^+^Foxp3^+^ Treg differentiation and IL-10 production in wild-type (WT) and HSF1^fl/fl^-CD4^cre^ T cells. Flow cytometry analysis (Figure 1a,b) shows that celastrol significantly increases the percentage of CD4^+^Foxp3^+^ Tregs at 100 nM, whereas higher concentrations (200 nM) result in a decline, consistent with reduced Treg fitness at elevated doses [34]. Western blot analysis (Figure 1c) demonstrates celastrol-induced HSF1 cytoplasmic activation and nuclear translocation, suggesting its role in transcriptional regulation. Western blot original images can be found in Supplementary Materials. Luciferase assays (Figure 1d,e) confirm that celastrol enhances HSF1 binding to the heat shock element (HSE) promoter region and increases Foxp3 promoter activation. However, HSF1-deficient Tregs display reduced Foxp3 expression overall, but celastrol still produces a modest and statistically significant increase, albeit markedly lower than the response observed in WT Tregs (Figure 1f). In addition, HSF1-deficient Tregs exhibit reduced IL-10 production (Figure 1g,h), suggesting that celastrol-induced Treg functionality is partly HSF1-dependent. These findings indicate that HSF1 activation is critical for celastrol-mediated Treg differentiation and immunosuppressive function.

### 3.2. Celastrol Enhances Treg-Mediated Suppression in an HSF1-Dependent Manner

**Figure 2:**
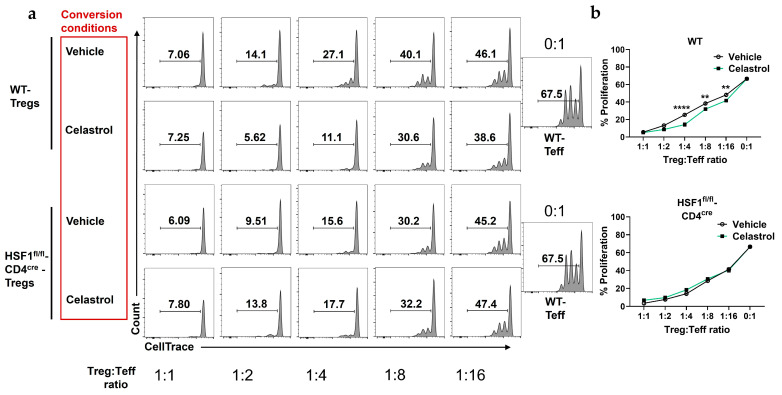
Celastrol promotes Treg-mediates suppression of effector T cells (Teff) and IL-10 production. (**a**) Representative flow cytometry plots showing suppressive effect of a decreasing concentration of Treg (CD4^+^CD25^+^) cells with constant Teff (CD4^+^CD25^−^) cells amount from WT and HSF1^fl/fl^-CD4^cre^ mice splenocytes. Cells stained with CellTrace and counted by flow cytometry (n = 4). (**b**) Treg:Teff suppression assay showing enhanced suppressive capacity of Tregs on the proliferation of Teff cells following Celastrol treatment (n = 4). Results are presented as individual mice ± SEM. Statistical significance was determined using two-way ANOVA (**b**) followed by Tukey’s multiple comparison test (** *p* < 0.01, **** *p* < 0.0001).

First, to determine whether celastrol independently affects Teff in the absence of Tregs, we treated purified Teff cells with increasing concentrations of celastrol (0, 50, 100, 200, and 300 nM). Our result showed that celastrol reduced Teff viability in a dose-dependent manner (Figure S1). Next, to evaluate the functional capacity of Tregs, we performed a Treg:Teff suppression assay using Tregs from WT and HSF1^fl/fl^-CD4^cre^ mice after in vitro conversion in the presence or absence of celastrol. These converted Tregs were then co-cultured with CellTrace-labeled Teff cells. Flow cytometry histograms (Figure 2a) illustrate Teff proliferation across Treg:Teff ratios under vehicle or 100 nM celastrol treatment. In WT Tregs, celastrol markedly enhanced suppressive activity, evidenced by a significant reduction in Teff proliferation. Quantification of proliferation (Figure 2b) confirmed that celastrol-treated WT Tregs suppressed Teff expansion more effectively than vehicle controls. In contrast, HSF1-deficient Tregs (HSF1^fl/fl^-CD4^cre^) failed to exhibit this celastrol-induced enhancement, showing no difference between vehicle- and celastrol-treated groups. Together, these findings demonstrate that celastrol enhances Treg-mediated suppression in an HSF1-dependent manner, indicating that HSF1 is required for celastrol-induced Treg functionality.

### 3.3. Celastrol Protects Against Colitis by Enhancing Treg-Mediated IL-10 Production and Reducing Inflammation

**Figure 3:**
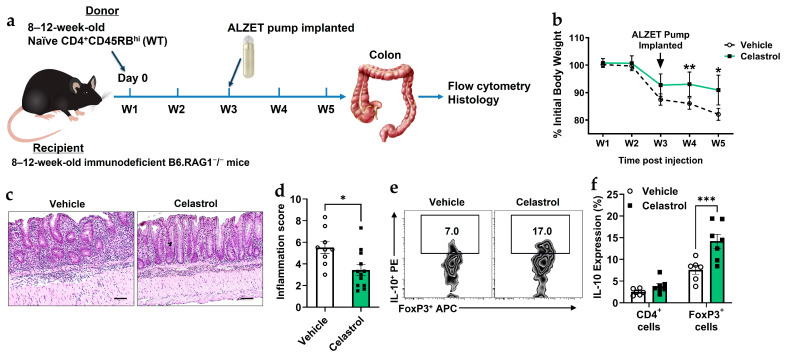
Celastrol protects against murine colitis by enhancing Treg functionality. (**a**) Schematic of experimental timeline. (**b**) Body weight change following adoptive transfer and treatment with Vehicle or 100 nM Celastrol over a five-week period (W1–W5) (n = 10). (**c**) Representative hematoxylin and eosin (H&E) staining of colonic tissues from Vehicle- and 100 nM Celastrol-treated mice. Scale bar = 50 µm (n = 10). (**d**) Quantification of inflammation scores in colonic tissues from Vehicle- and Celastrol-treated mice (Vehicle n = 10, Celastrol n = 12). (**e**) Representative flow cytometry plots showing CD4^+^Foxp3^+^IL-10^+^ Treg populations in colonic tissues from Vehicle- and Celastrol-treated mice (n = 5). (**f**) Quantification of IL-10 expression (from (**e**)) in total CD4^+^ T cells and CD4^+^Foxp3^+^ Tregs following Vehicle or Celastrol treatment (n = 5). Results are presented as individual mice ± SEM. Statistical significance was determined using two-way ANOVA (**d**,**f**) followed by Tukey’s multiple comparison test, and Student’s *t*-test (**d**) (* *p* < 0.05, ** *p* < 0.01, *** *p* < 0.001). Absolute number of cells shown in Figure S3B.

A recent study demonstrated that celastrol protects mice from developing colitis [41]. In this study, we used an adoptive transfer colitis model in B6 RAG1^−^/^−^ mice to investigate the role of Tregs in the presence or absence of celastrol. Naïve CD4^+^CD45RB^hi^ (WT) were introduced to these immunodeficient mice, which lack B and T cells. To evaluate the therapeutic effect of celastrol in a murine colitis model, we analyzed body weight changes, histological inflammation, and IL-10 expression in response to the induced colitis (Figure 3a). Body weight measurements (Figure 3b) show that celastrol treatment prevents weight loss compared to vehicle-treated mice, particularly after pump implantation. Histological analysis of colonic tissues (Figure 3c,d) reveals a significant reduction in inflammation scores in celastrol-treated mice. Moreover, flow cytometry analysis (Figure 3e,f) demonstrates that celastrol increases IL-10 production in FoxP3^+^ Tregs, indicating an enhanced immunosuppressive function of Tregs. Collectively, these findings suggest that celastrol protects against colitis by increasing Treg-mediated IL-10 production and reducing intestinal inflammation.

### 3.4. Celastrol Reduces Spontaneous Ileitis and Modulates Immune Cell Populations in TNF^ΔARE^ Mice

**Figure 4:**
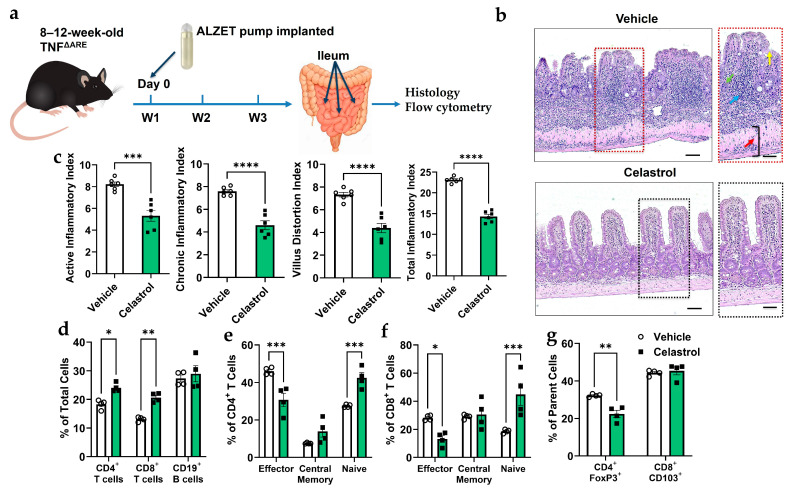
Celastrol reduces inflammation in the ileum of 20-week-old TNF^ΔARE^ mice. (**a**) Schematic of experimental timeline. (**b**) Representative images of ileal mucosa with or without celastrol treatment. Blue arrow = inflammatory cell infiltration, green arrow = destroyed crypt cells, yellow arrow = distorted villi, and red arrow = enlarged muscularis layer. Scale bar = 50 µm (n = 6). (**c**) Inflammation score describing structural changes that were improved with celastrol treatment (n = 6). (**d**) Flow cytometry analysis of total number of cells in percentage (fom lamina propria) shows that celastrol increases CD4^+^ and CD8^+^ T cell subpopulations while maintaining CD19^+^ B cell levels (n = 4). (**e**) Flow cytometry analysis showing that celastrol treatment significantly decreases effector CD4^+^ T cells while increasing naive CD4^+^ T cells, with no significant effect on central memory T cells (n = 4). (**f**) Flow cytometry analysis showing that celastrol treatment significantly decreases effector CD8^+^ T cells while increasing naive CD8^+^ T cells, with no significant effect on central memory T cells (n = 4). (**g**) Flow cytometry analysis showing that celastrol treatment significantly decreases the percentage of CD4^+^Foxp3^+^ regulatory T cells (out of the CD4^+^ parent cells) while having no significant effect on CD8^+^CD103^+^ T cells (out of the CD8^+^ parent cells) (n = 4). Results are presented as individual mice ± SEM. Statistical significance was determined using two-way ANOVA (**d**,**e**,**f**,**g**) followed by Tukey’s multiple comparison test, and Student’s *t*-test (**a**) (* *p* < 0.05, ** *p* < 0.01, *** *p* < 0.001, **** *p* < 0.0001). Absolute number of cells shown in Figure S3C.

The TNF^ΔARE^ model is a well-established system for studying spontaneous ileitis in mice [39]. To assess the effects of celastrol on TNF^ΔARE^ mice (Figure 4a), we evaluated intestinal inflammation and immune cell composition. Histological scoring (Figure 4b) reveals that celastrol significantly reduces active and chronic inflammation, villus distortion, and total inflammatory index. H&E staining (Figure 4c) confirms reduced inflammatory infiltration and improved intestinal architecture in celastrol-treated mice compared to Vehicle controls. Furthermore, flow cytometry analysis (Figure 4d–g) demonstrates that celastrol treatment decreases CD4^+^ and CD8^+^ effector T cells while increasing naïve CD4^+^ and CD8^+^ T cell populations. Additionally, celastrol significantly reduces CD4^+^Foxp3^+^ Tregs, but does not alter CD8^+^CD103^+^ T cells. These findings suggest that celastrol alleviates inflammation in TNF^ΔARE^ mice by modulating immune cell composition and reducing pathogenic effector T cells.

## 4. Discussion

Celastrol, a bioactive compound derived from the thunder god vine (*Tripterygium wilfordii*), exhibits potent anti-inflammatory effects through multiple molecular mechanisms [42]. One key pathway involves the activation of Heat Shock Factor 1 (HSF1) (as demonstrated in the present study), a transcription factor that upregulates the expression of heat shock proteins (HSPs), particularly HSP70 [43]. These proteins act as molecular chaperones that stabilize protein folding and suppress pro-inflammatory signaling pathways, such as NF-κB [44]. In addition to HSF1 activation, celastrol directly inhibits the IκB kinase (IKK) complex, thereby preventing the phosphorylation and degradation of IκBα, which in turn retains NF-κB in the cytoplasm and reduces the transcription of pro-inflammatory cytokines [45]. Celastrol also modulates oxidative stress by enhancing antioxidant responses, further contributing to its anti-inflammatory action [46]. Through this combination of heat shock response activation, NF-κB pathway inhibition, and oxidative stress reduction, celastrol exerts a broad and multi-targeted suppression of inflammation. Since HSPs are strongly shaped by microbial and nutrient-derived signals, it is also possible that celastrol may alter the gut microbiota and thereby indirectly influence Treg stability or inflammatory tone [47,48]. Although this mechanism cannot be excluded, our current study did not include microbiota profiling or manipulation; future investigations using 16S rRNA sequencing, antibiotic depletion, or fecal-transfer approaches will be essential to determine whether any component of celastrol’s immunoregulatory effects is microbiota-mediated. Further, these additional mechanisms likely function synergistically with the HSF1-Treg pathway, underscoring HSF1 activation as the likely upstream regulator of celastrol’s immunomodulatory action.

In the current study, we demonstrate that celastrol enhances Treg functionality and ameliorates inflammation in murine colitis and spontaneous ileitis (TNF^ΔARE^ mice) through an HSF1-dependent mechanism. Our results provide evidence that celastrol promotes the differentiation and suppressive function of CD4^+^Foxp3^+^ Tregs, as indicated by increased Foxp3 expression and IL-10 production. Importantly, the effects of celastrol were abolished in HSF1-deficient Tregs, indicating that HSF1 is required for celastrol-induced Treg activation. These findings align with previous reports highlighting the role of HSF1 as a transcriptional regulator of immune homeostasis and stress responses [20]. Therefore, our data suggest that pharmacological activation of HSF1 by celastrol enhances Treg-mediated immunosuppression, thereby alleviating inflammation in colitis and spontaneous ileitis models.

Mechanistically, our Treg:Teff suppression assays demonstrate that celastrol-treated WT Tregs exhibit enhanced suppressive capacity, significantly reducing effector T cell (Teff) proliferation. However, this suppressive function was absent in HSF1^fl/fl^-CD4^cre^ mice, further confirming that HSF1 is a key mediator of Treg function. These findings are consistent with prior studies showing that HSF1 promotes the stability of Foxp3 expression and enhances Treg immunosuppressive activity [20]. Similarly, other studies corroborate the anti-inflammatory effect of celastrol in psoriatic inflammation by modulating Th17 cell population [49] and rheumatoid arthritis [50]. Additionally, our luciferase assay results confirm that HSF1 directly binds to the heat shock element (HSE) in the Foxp3 promoter, driving its transcriptional activation as previously shown [20]. Future ChIP-based analyses will be essential to directly confirm that celastrol enhances HSF1 occupancy at this promoter region, thereby strengthening the mechanistic link suggested by our luciferase data. Furthermore, a plethora of studies concur that celastrol-mediated activation of HSF1 could have implications in reducing pro-inflammatory cytokines such as IL-1β [51], TNFα [52], and IL-6 [53], in spite of the fact that some studies have shown that HSF1 may negatively influence expression of IL-1β and TNFα in IBD [51,52]. The net of our findings supports the hypothesis that celastrol augments Treg function through an HSF1-dependent pathway, which may have therapeutic potential for inflammatory diseases.

Regarding the colitis model, while our findings strongly support an HSF1-dependent mechanism, we acknowledge important limitations in our preliminary adoptive-transfer model using HSF1-deficient CD45RB^hi^ T cells. HSF1^−/−^ T cells inherently drive more severe baseline intestinal inflammation, complicating interpretation of celastrol responsiveness independent of disease intensity. Moreover, because celastrol influences multiple immune and stromal cell types, this model does not provide a completely controlled comparison for isolating Treg-specific HSF1 requirements. Although a whole-body HSF1 knockout would provide clearer mechanistic resolution, its partial embryonic lethality in C57BL/6 mice makes such studies technically challenging. For these reasons, our ex vivo assays served as the primary mechanistic system, demonstrating that celastrol enhances Treg function only when HSF1 is present, reinforcing the centrality of HSF1 in mediating celastrol’s immunoregulatory effects.

Beyond Treg enhancement and consistent with previous studies, celastrol exhibited potent anti-inflammatory effects in murine colitis and TNF^ΔARE^ mice [20]. Together, these models represent distinct but complementary inflammatory mechanisms, i.e., TNF^ΔARE^ dominated by dysregulated innate cytokine production and adoptive transfer colitis by pathogenic adaptive immune activation that allows robust validation of our findings across two mechanistically different IBD settings. Histological scoring of colonic and ileal tissues revealed that celastrol significantly reduced inflammatory indices, villus distortion, and immune cell infiltration. Furthermore, flow cytometry analysis revealed a significant shift in T cell subset composition, with celastrol reducing effector T cells (CD4^+^ and CD8^+^) while increasing naïve T cells. These changes are indicative of celastrol’s ability to dampen excessive immune activation and restore immune balance. Interestingly, while celastrol significantly reduced CD4^+^Foxp3^+^ Tregs, its ability to enhance IL-10 production suggests a functional shift rather than depletion. These findings suggest that celastrol may exert its protective effects not only by increasing Treg function but also by modulating the overall immune landscape within inflamed tissues.

It appears that celastrol exerted model-specific effects on Treg abundance and function, a pattern consistent with the distinct inflammatory and metabolic environments of the WT (Figure 1 and Figure 2), colitis (Figure 3), and TNF^ΔARE^ ileitis (Figure 4) settings. In WT mice, where Tregs remain stable and experience minimal stress, celastrol-induced HSF1 activation enhances FoxP3 stability and Treg survival, leading to increased Treg frequency consistent with both our current findings and prior work from our laboratory [20]. In adoptive transfer colitis, celastrol primarily restores Treg functionality as reflected by increased IL-10 production [54]. This was also further strengthened by clinical experiments [55]. Nevertheless, celastrol was also shown to ameliorate colitis in an IL-10-independent manner [56]. Moreover, although we attempted adoptive transfer of Tregs differentiated ex vivo in the presence of celastrol, the regulatory effects were not durable after transfer, as Tregs rapidly lost celastrol-dependent enhancements without continued exposure, making this approach unsuitable for cleanly isolating Treg-intrinsic effects in vivo.

By contrast, the TNF^ΔARE^ ileitis model is characterized by chronic TNF-driven ER stress and an overexpanded, unstable Treg compartment [15,57]. As previously reported, TNF^ΔARE^ mice display increased numbers of CD4^+^ T cells and FoxP3^+^ Tregs as a consequence of severe intestinal inflammation; however, these Tregs exhibit functional defects, including reduced IL-10 secretion and impaired suppressive capacity in vitro [37]. In line with these observations, we routinely find that when inflammation decreases in this model, the abnormally expanded Treg population contracts, yet the remaining Tregs show improved IL-10 production. Thus, as celastrol alleviates inflammation and reduces ER stress, this stress-induced Treg expansion contracts, producing an apparent decrease in FoxP3^+^ Tregs despite improved IL-10-mediated regulatory activity [32,58]. Thus, celastrol’s effects reflect the underlying biology of each model: enhancement of homeostatic Tregs in WT mice, functional rescue in colitis, and normalization of an aberrantly expanded Treg pool in TNF^ΔARE^ ileitis.

## 5. Conclusions

Taken together, our findings uniquely highlight celastrol as a potential immunomodulatory therapy for autoimmune and inflammatory diseases. By targeting HSF1, celastrol enhances Treg-mediated suppression, restores immune homeostasis, and reduces colonic and ileal inflammation. Given the significant T cell subset alterations and suppression of Teff proliferation, future studies should investigate whether celastrol can be used as a standalone treatment or in combination with existing immunotherapies. Additionally, further mechanistic studies are warranted to explore celastrol’s effects on other immune pathways and its potential therapeutic applications beyond colitis and spontaneous ileitis. These findings underscore the translational potential of celastrol as an HSF1-targeting therapy for immune-mediated disorders, including IBD.

## Data Availability

The data underlying this article are available in the article and in its online Supplementary Material and will be shared on reasonable request to the corresponding author.

## Supplementary Materials

The following supporting information can be downloaded at: https://www.mdpi.com/article/10.3390/biom16010062/s1, Figure S1: Dose-dependent inhibition of lymphocyte by celastrol; Figure S2: Gating strategies for flow cytometry data; Figure S3: Absolute number of parent cells for flow cytometry graphs. And Western blot original images.

## Abbreviations

The following abbreviations are used in this manuscript:

IBD	Inflammatory Bowel Disease
HSR	Heat Shock Response
HSF1	Heat Shock factor 1
Tregs	Regulatory T cells
TNFα	Tumor Necrosis Factor-alpha
JAK	Janus Kinase
IPEX	Immune Dysregulation, polyendocrinopathy, Enteropathy X-linked
HSE	Heat Shock Response Element
TCR	T Cell Receptor
MD2	Myeloid Differentiation Factor 2
FBS	Fetal Bovine Serum
RBC	Red Blood Cell
IACUC	Institutional Animal Care and USE Committees
SPF	Specific Pathogen Free
WT	Wild Type
H&E	Hematoxylin and Eosin
LP	Lamina Propria
ELISA	Enzyme-Linked Immunosorbent Assay
