# Supplementary figures and images for: Celastrol Activates HSF1 to Enhance Regulatory T Cells Function and Ameliorate Intestinal Inflammation
Source: Biomolecules. 2025 Dec 31;16(1):62. doi: 10.3390/biom16010062 (PMC12839249; doi:10.3390/biom16010062)

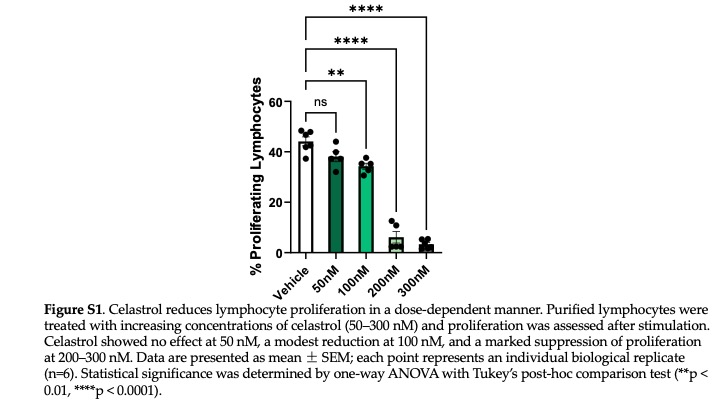

Supplement: Supplementary file 1 [file biomolecules-16-00062-s001.zip › Figure S1.jpeg]

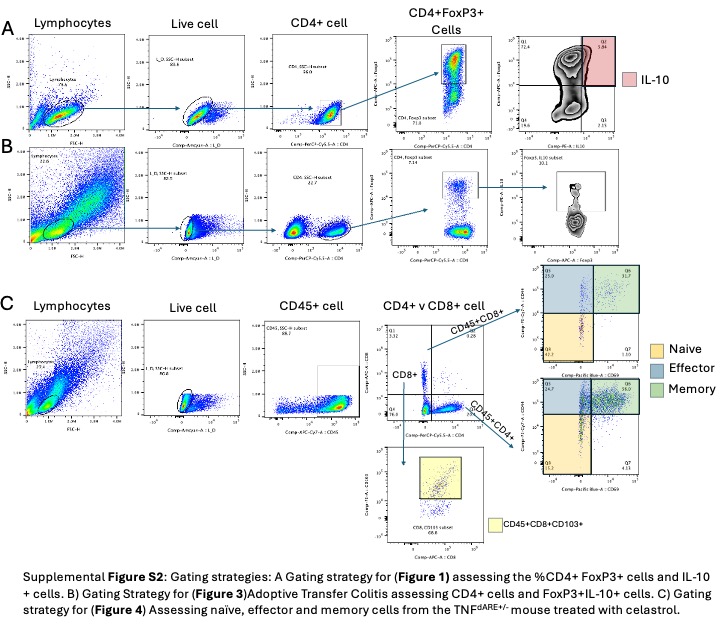

Supplement: Supplementary file 1 [file biomolecules-16-00062-s001.zip › Figure S2.jpeg]

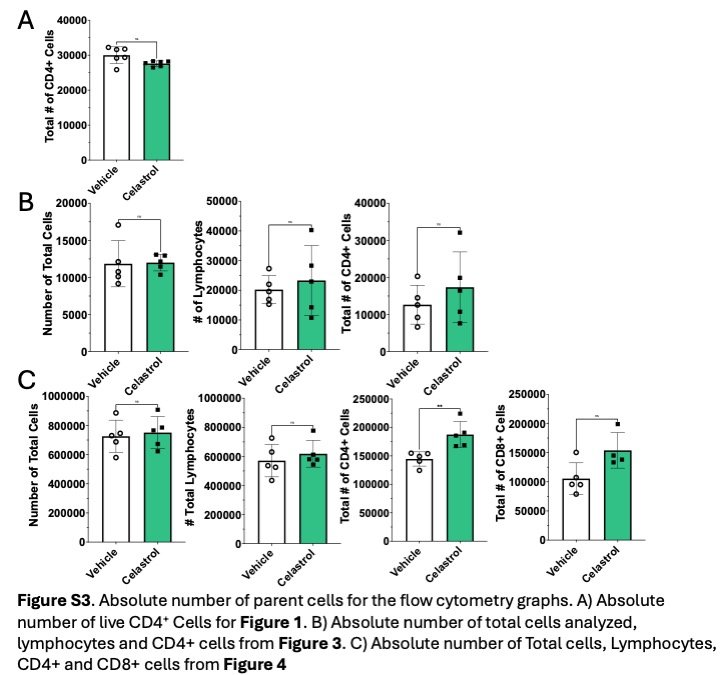

Supplement: Supplementary file 1 [file biomolecules-16-00062-s001.zip › Figure S3.jpeg]

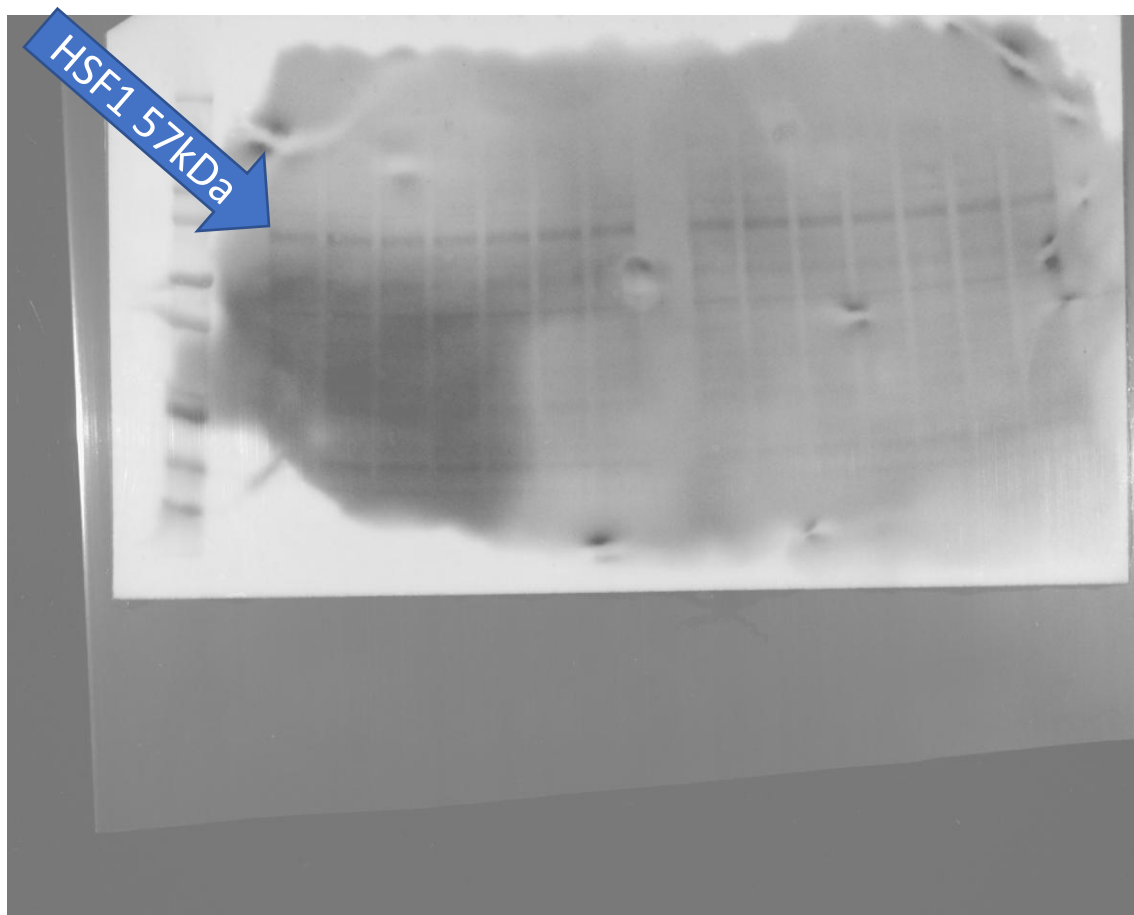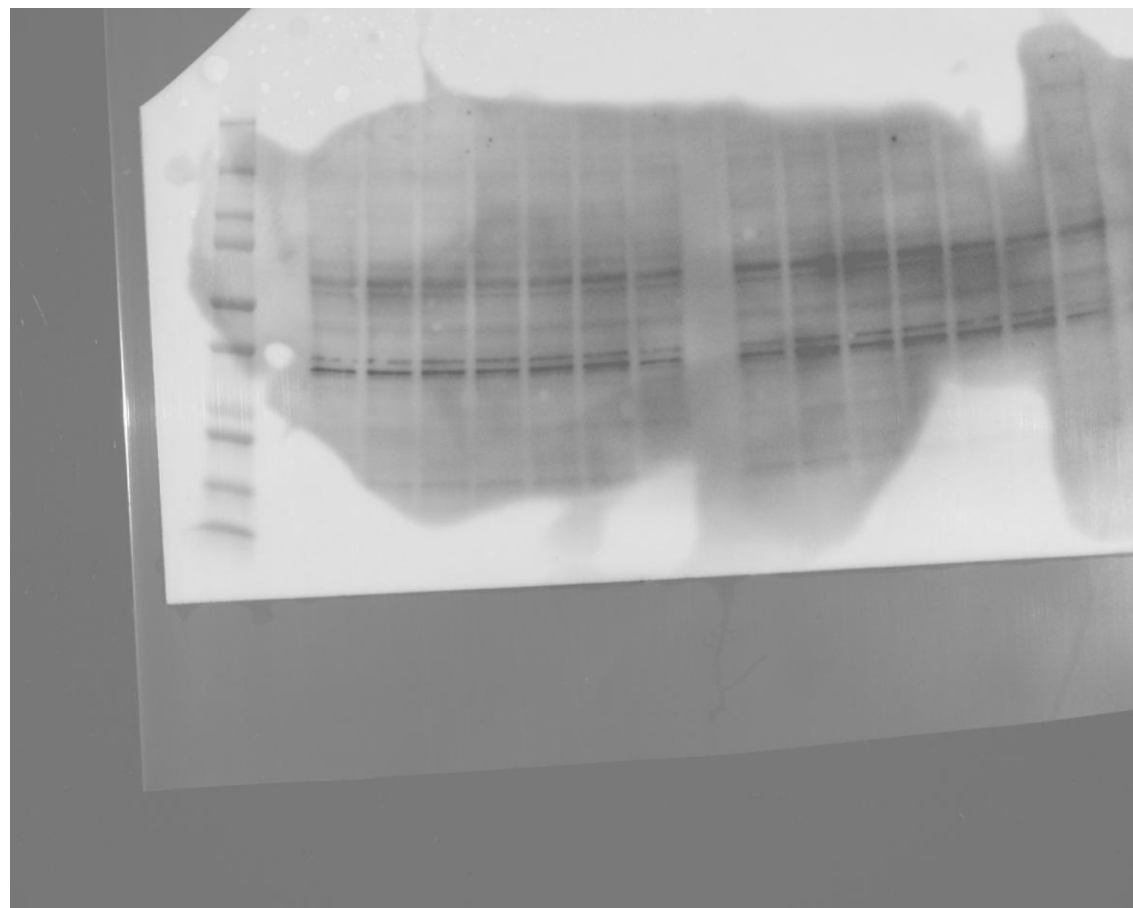

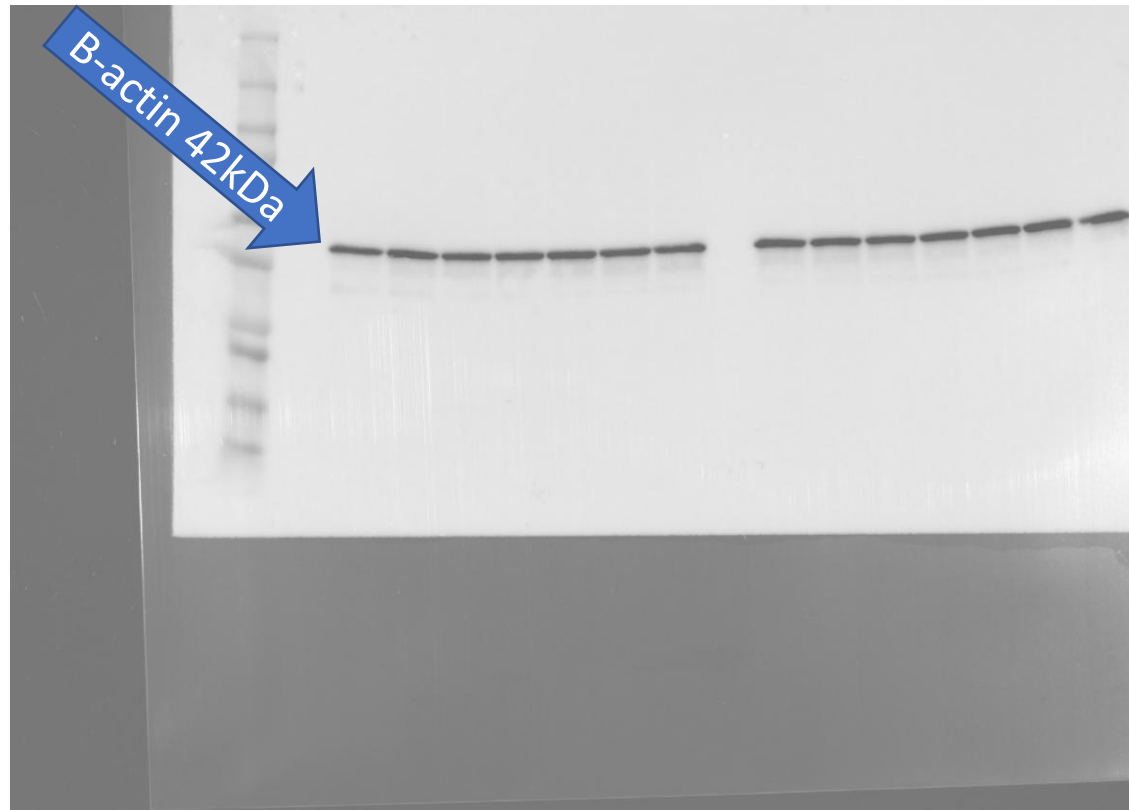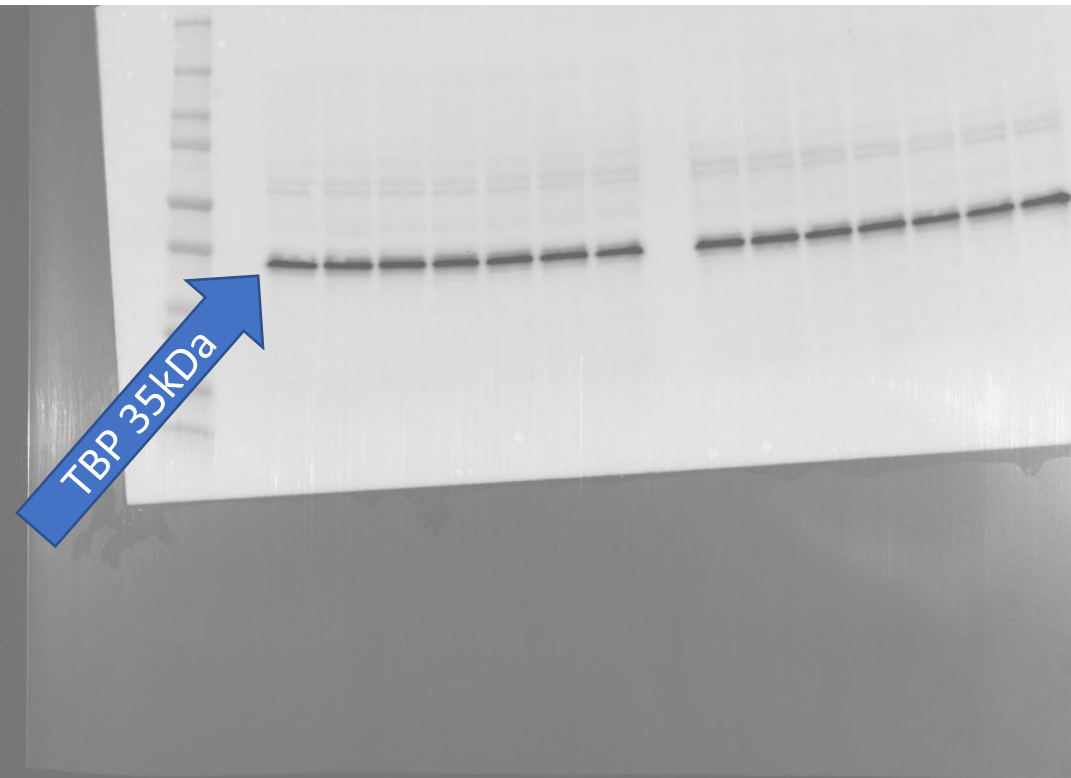

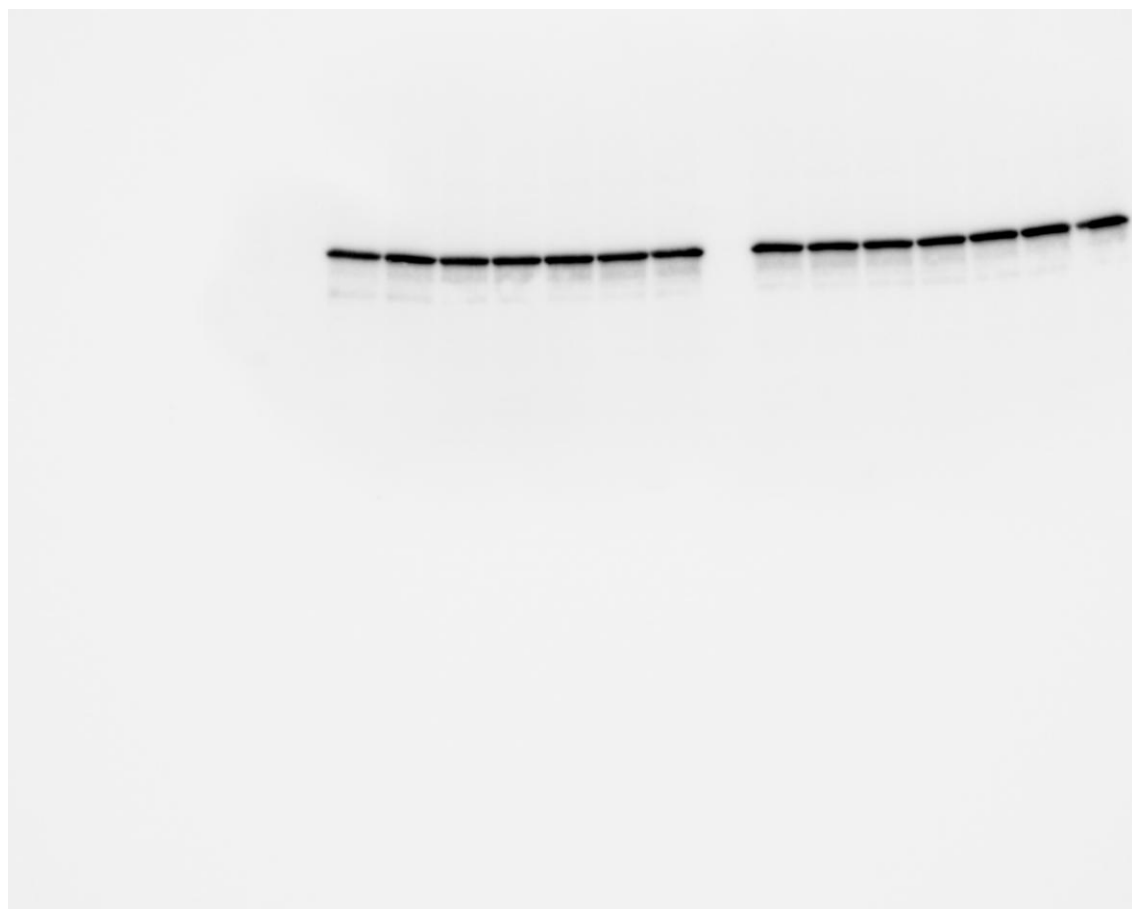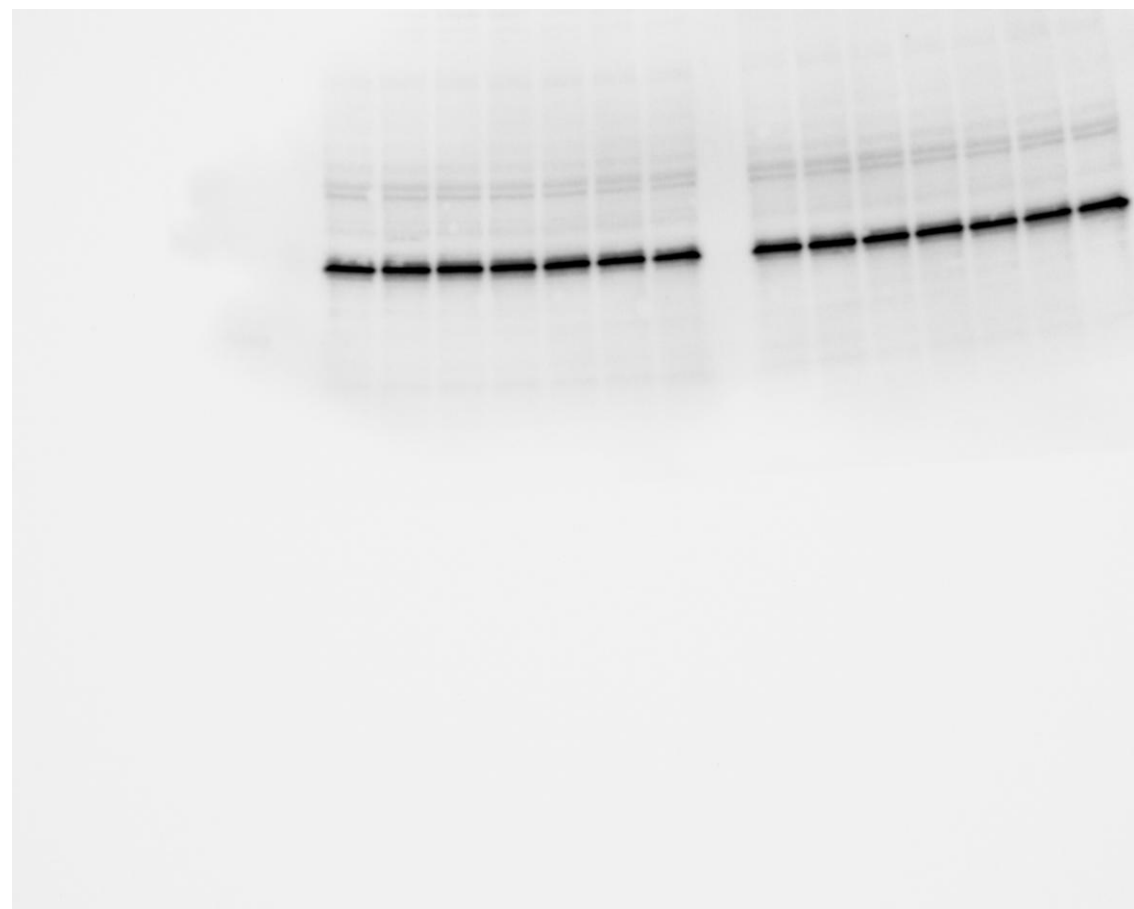

Supplement: Supplementary file 1 [file biomolecules-16-00062-s001.zip › Western blot original images.pdf]
